# Comparing the Efficacy of Desidustat Versus Erythropoietin in the Management of Anemia in Patients With Dialysis-Naïve Chronic Kidney Disease: A Randomized Open-Label Trial

**DOI:** 10.7759/cureus.103690

**Published:** 2026-02-15

**Authors:** Hiramani Rabha, Saif Quaiser, Syed Shariq Naeem, Waseem Rizvi

**Affiliations:** 1 Pharmacology, Jawaharlal Nehru Medical College, Aligarh Muslim University, Aligarh, IND; 2 Medicine, Jawaharlal Nehru Medical College, Aligarh Muslim University, Aligarh, IND

**Keywords:** anemia in ckd, chronic kidney disease (ckd), desidustat, hif-phi, non-dialysis

## Abstract

Background: Anemia is a serious complication of chronic kidney disease (CKD). The current standard of care for anemia of CKD is erythropoiesis-stimulating agents (ESAs). However, ESAs have several limitations, such as compliance issues, high cost, cardiovascular risks, and potential immunogenicity. Desidustat is a novel oral hypoxia-inducible factor prolyl hydroxylase inhibitor (HIF-PHI) that enhances endogenous erythropoietin synthesis and iron utilization.

Methods: This open-label, randomized, prospective, non-inferiority trial included 60 dialysis-naïve CKD patients with baseline hemoglobin of ≤9 g/dL and adequate iron stores who were randomized (1:1) to receive either desidustat orally or erythropoietin subcutaneous injection for six months, once every two weeks.

Results: Desidustat showed non-inferiority to erythropoietin in increasing and maintaining hemoglobin levels within the target range over six months. The mean hemoglobin increased in the desidustat arm from baseline 7.99±0.73 to the estimated marginal mean (EMM) of 10.01±0.21 (95% CI: 9.59-10.4), while the mean hemoglobin in the erythropoietin arm increased from baseline 7.68±0.84 to the EMM of 9.89±0.21 g/dL (95% CI: 9.46-10.3). The percentage of hemoglobin responders was higher with desidustat (15 (57.69%)) compared to erythropoietin (12 (48%)). Both treatments showed comparable improvements in hematological parameters and iron profiles. Desidustat had a greater improvement in quality of life score (Chronic Kidney Disease-Anemia Questionnaire (CKD-AQ) compared to erythropoietin at each time interval, reflecting lower symptom burden. From baseline to six months, an increased level of growth differentiation factor-15 (GDF-15) and a decreased level of interleukin 6 (IL-6) were noted in both arms. No correlation was found between hemoglobin difference, biochemical parameters, and biomarkers.

Conclusion: Desidustat was non-inferior to erythropoietin in treating anemia of dialysis-naïve CKD patients. Desidustat offers a safe and effective alternative to ESAs with better compliance.

## Introduction

Chronic kidney disease (CKD) is a widespread chronic illness, ranking as the 12th leading cause of death worldwide in 2017 [1], affecting over 800 million people globally as of 2022 [2]. It increases morbidity and mortality and significantly diminishes the quality of life (QoL).

Anemia is a common complication of CKD, with India having the highest percentage of anemic individuals globally [3,4]. The most frequent cause of anemia in hospitalized patients is anemia of chronic disease, while the most prevalent form is iron deficiency anemia (IDA) [5]. The incidence increases with age, affecting approximately 77% of the elderly and significantly impacting the QoL and survival rates in chronically ill patients [6]. In non-dialysis CKD patients, the prevalence of anemia is as high as 60%.

The most commonly used treatments for anemia of CKD are erythropoiesis-stimulating agents (ESAs), iron supplements, and blood transfusions [7]. However, a newer class of drugs, hypoxia-inducible factor prolyl hydroxylase inhibitor (HIF-PHI), has shown promising results in treating anemia associated with CKD, regardless of the dialysis status [5]. Desidustat is an oral HIF-PHI, approved in India on March 7, 2022, for treating anemia with CKD, regardless of dialysis status [8]. HIF-PHIs promote erythropoiesis by inhibiting HIF-prolyl hydroxylase activity [9]. Desidustat also lowers hepcidin expression by modulating the hepcidin-ferroportin axis, which increases iron availability and supports erythroid development. Furthermore, it decreases the levels of inflammatory markers such as IL-6 and IL-1β and improves erythropoietin (EPO) sensitivity, thereby reducing anti-EPO antibodies [10].

ESAs, while effective, are associated with several adverse effects, including seizures, hypertension, clotting issues during dialysis, increased mortality in patients with malignancies, and disease progression in cancer patients. In some patients, due to hyporesponsiveness to EPO and resistance due to anti-EPO antibodies linked to pure red cell aplasia (PRCA), ESA therapy is ineffective. High doses of ESAs have been associated with an increased risk of mortality, cardiovascular events, and hypertension [11]. Further, ESAs may exert extra-hematopoietic actions that influence arterial blood pressure, vascular endothelium, and the coagulation system. Also, EPO activates pro-inflammatory cytokines, contributing to cardiovascular remodeling and inflammation. It increases platelet count, coagulation, and thrombotic complications [12].

Erythropoietin (epoetin-α), a commonly used ESA, is typically administered subcutaneously and often requires multiple doses, which may pose compliance challenges for patients. In contrast, desidustat is an orally administered tablet that serves as a convenient alternative to injectable ESAs. It will enhance patient compliance due to the ease of administration. It is also non-inferior to epoetin-α in CKD stage V patients on dialysis [13]. It improves anemia in the erythropoietin hyporesponsive state [10]. Anti-inflammatory and cardioprotective activity can also be an additional benefit in CKD patients.

Moreover, there is no study comparing the efficacy and safety profile of desidustat vs. erythropoietin in CKD stage III-V patients with anemia not on dialysis. There are also no studies correlating the inflammatory markers with desidustat in anemia of CKD stage III-V patients not on dialysis. Therefore, our study was conducted to evaluate the efficacy and safety profile of desidustat vs. erythropoietin in anemia of CKD stage III-V in dialysis-naïve patients. It also explored the impact of desidustat on QoL and correlated the hemoglobin difference from baseline to six months with biochemical parameters and biomarkers.

## Materials and methods

Study design 

This study was a randomized, open-label, parallel-arm, prospective, and non-inferiority trial conducted at Jawaharlal Nehru Medical College and Hospital, Aligarh Muslim University, Aligarh, India, to evaluate the efficacy and safety profile of desidustat vs. erythropoietin in anemia of CKD stages III-V in dialysis-naïve patients. Enrolled patients were randomized by fixed block randomization by the treating physician, at a ratio of 1:1, to either desidustat or erythropoietin arms, according to the table generated in RStudio (Posit PBC, Boston, Massachusetts, United States) using "blockrand" and "randomizeR" packages. The study was conducted on adult patients of either sex with anemia in dialysis-naïve CKD (stages III-V). Ethical clearance for the study protocol was obtained from the Institutional Ethics Committee of Jawaharlal Nehru Medical College and Hospital, Faculty of Medicine, Aligarh Muslim University (approval number: IECJNMC/867). The study was prospectively registered in the Clinical Trials Registry-India (CTRI) (CTRI number: CTRI/2023/05/052681). The patients were informed about all possible and expected advantages and disadvantages of the study. Before enrolling in the study, written and informed consent was obtained from all patients. 

Sample size calculation

Sample size estimation was done using the following formula: $\mathrm{n}=(2\times(\mathrm{Z}_{\mathrm{1-&alpha;}}+\mathrm{Z}_{\mathrm{1-&beta;}})^{2}\times\mathrm{\sigma}^{2})/\mathrm{&Delta;}^{2}$. Here, $\mathrm{Z}_{\mathrm{1-&alpha;}}$ is the Z value for one-sided alpha (0.025) ≈ 1.96, $\mathrm{Z}_{\mathrm{1-&beta;}}$ is the Z value for power 80% ≈ 0.84, σ is the SD of hemoglobin change = 1.3 g/dL [14], and Δ is the non-inferiority margin = 1 g/dL. Hence, the sample size (n) is 27 in each arm. With a 10% dropout rate, the sample size (n) becomes 30 in each arm. Since there are 30 patients in each arm, i.e., desidustat and erythropoietin arm, a total of 60 patients were enrolled.

Inclusion criteria

**Figure 1:**
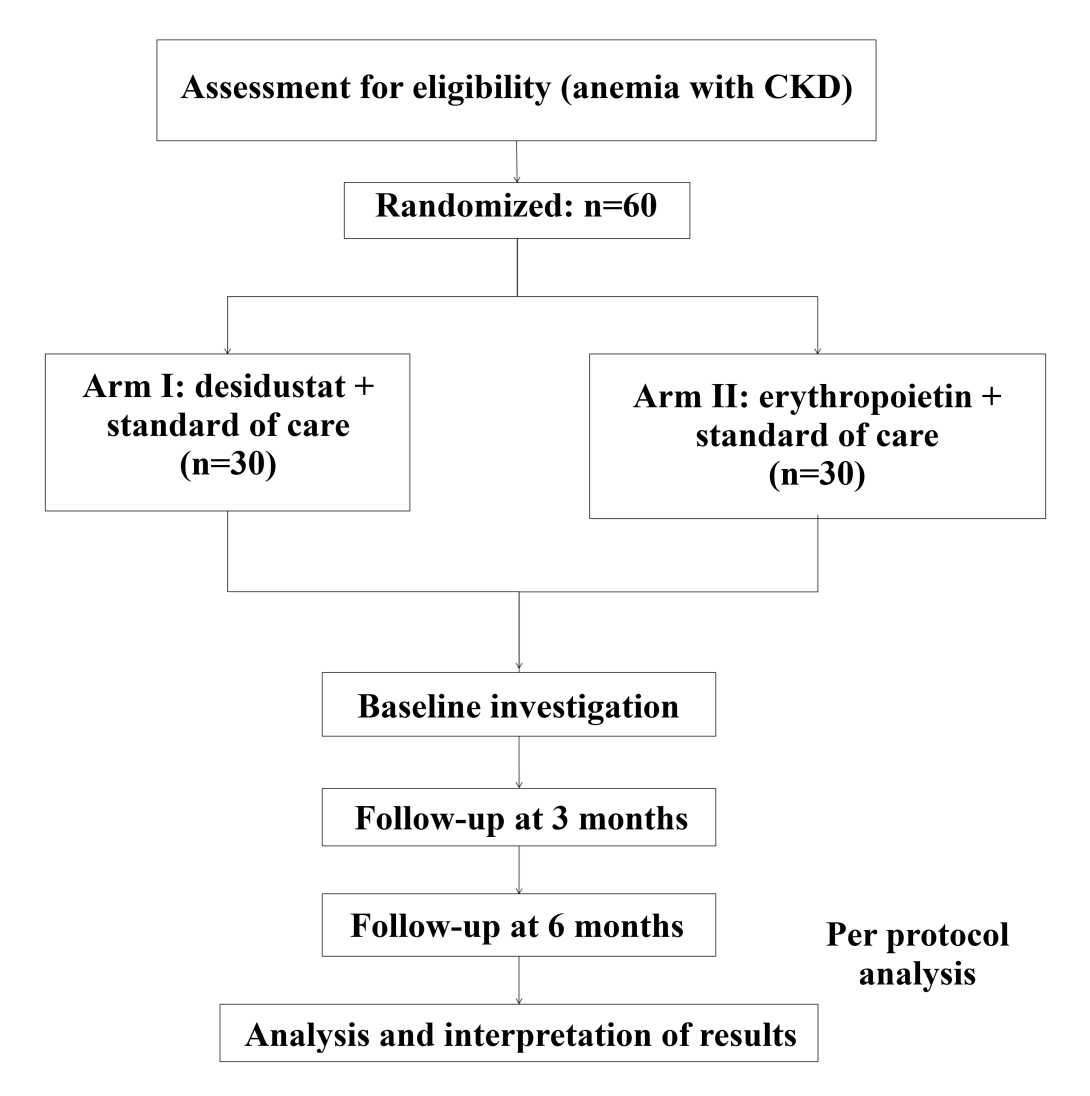
Flowchart of methodology CKD: chronic kidney disease

The study included adult patients of either sex with anemia (hemoglobin ≤9 g/dL) with CKD stages III-V not on hemodialysis or peritoneal dialysis, with adequate iron stores (serum ferritin ≥100 ng/mL, transferrin saturation (TSAT) >20%), and patients agreeing to give informed consent (Figure 1).

Exclusion criteria

Patients were excluded if they had hyperkalemia, history of hematological disorders (viz., uncontrolled autoimmune hemolytic anemia, idiopathic thrombocytopenic purpura (ITP), thalassemia), prior blood transfusion within six weeks of enrollment in the study, electrocardiogram (ECG) abnormalities during screening, allergy to desidustat or erythropoietin, cardiovascular disease (viz., uncontrolled arrhythmia, congestive heart failure), infectious diseases (active hepatitis, active tuberculosis), and pregnant and breastfeeding women (Figure 1).

Outcome measures

The primary outcome measure was the difference between the level of change in hemoglobin in the desidustat vs. erythropoietin arm over six months. Secondary outcome measures included the change in QoL score assessed by the Chronic Kidney Disease-Anemia Questionnaire (CKD-AQ) [15], the number of hemoglobin responders (defined as achievement of target level of 10-12 g/dL and post-treatment increase of more than 1 g/dL in hemoglobin by six months), and the correlation of hemoglobin difference from baseline to six months with biochemical parameters and biomarkers.

Trial procedures

All the patients were subjected to detailed clinical history, thorough examination, and necessary routine baseline evaluation, including complete blood count, iron profile, renal function test, serum electrolytes, serum albumin, and liver function test. Diagnosis of anemia of CKD was made as per the Kidney Disease: Improving Global Outcomes (KDIGO) guideline. All randomized patients who completed the treatment and had not violated the protocol were included in the per-protocol (PP) population.

For this prospective study, 60 patients with anemia of CKD stage III-V non-dialysis were randomized into two arms. In one arm (n=30), desidustat 100 mg was given orally thrice a week for six months for the correction of anemia, and in the second arm (n=30), erythropoietin injection 0.75 ug/kg subcutaneously was given once every two weeks for six months, based on a previous study comparing desidustat and epoetin alpha [13]. Patients were also given oral or intravenous iron therapy depending on iron status along with standard of care. All patients were recommended to take a renal diet and water intake of 1.5 liters per day (Figure 1).

Safety analysis

All adverse events reported by patients or identified by the investigator were documented at each visit. Safety assessment was carried out using Naranjo's Adverse Drug Reaction Probability Scale [15] and Adverse Drug Reaction Severity Assessment Scale: Modified Hartwig and Siegel [16].

Statistical analysis

All data were recorded in the case record form and the Microsoft Excel spreadsheet program (Microsoft Corporation, Redmond, Washington, United States). The normally distributed variables were expressed as mean±standard deviation (SD). Categorical data were presented as proportions. For comparison of hematological parameters and biomarkers between both arms, analysis of covariance (ANCOVA) was used by controlling baseline data as a covariate and treatment as a fixed effect. Before testing ANCOVA, data was tested for normality of residual, homogeneity of variances, and independence of covariate and treatment, and violations were recorded. Hemoglobin responders in both arms were tested using the chi-squared test. Pearson's correlation was used to correlate the hemoglobin difference from baseline to six months with biochemical parameters and biomarkers. A p-value of less than 0.05 was considered statistically significant. The data analysis was done using IBM SPSS Statistics for Windows, Version 23.0 (IBM Corp., Armonk, New York, United States) and R (R Foundation for Statistical Computing, Vienna, Austria) using statistical packages (ggstatsplot, tidyverse, ggplot, emmeans, effectsize). The non-inferiority margin was considered from the previous studies as 1.0 [13].

## Results

Patient characteristics

**Figure 2:**
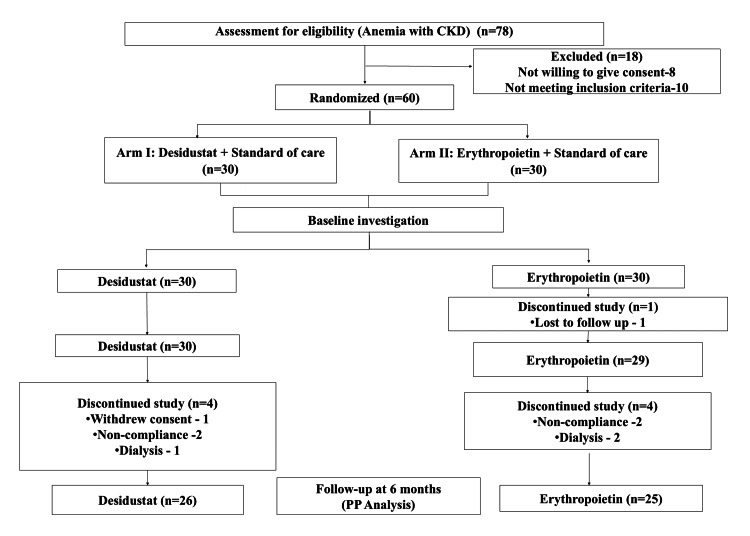
CONSORT diagram of the study CONSORT: Consolidated Standards of Reporting Trials; CKD: chronic kidney disease; PP: per protocol

Out of 78 patients assessed for eligibility for anemia with CKD, 18 were excluded due to inability to meet the inclusion criteria and unwillingness to give consent. A total of 60 patients with anemia of CKD stage III-V non-dialysis were randomized into two arms. Blood samples were collected for all randomized patients at baseline, three months, and six months. All randomized patients who completed the treatment and had not violated the protocol were included in the PP population. In the desidustat arm, 26 (86.7%) patients completed the study and were included in the PP population at six months. Four (13.3%) patients discontinued the study (withdrew consent: one; non-compliance: two; put on dialysis: one). In the erythropoietin arm, out of 30 patients, five (16.7%) patients were excluded from the study (lost to follow-up: one; non-compliance: two; put on dialysis: two); hence, 25 (83.3%) patients were included in the PP population at six months (Figure 2). Demographic profile and baseline characteristics were mentioned in Table 1.

**Table 1:** Demographic and baseline characteristics Values are presented as number or mean±standard deviation as appropriate. Standardized mean difference was calculated using Cohen's d, and the difference in means was divided by the pooled standard deviation. SBP: systolic blood pressure; DBP: diastolic blood pressure; BMI: body mass index; BSA: body surface area; CrCL: creatinine clearance; eGFR: estimated glomerular filtration rate; CKD: chronic kidney disease; SD: standard deviation

Parameters	Overall (n=60)	Desidustat (n=30)	Erythropoietin (n=30)	Standardized mean difference
	Mean±SD	Mean±SD	Mean±SD	
Age (years)	50.75±16.33	51.24±16.03	50.00±17.52	0.09
Gender (n)	Male=23; female=37	Male=12; female=18	Male=11; female=19	-0.07
Weight (kg)	60.88±12.74	61.47±10.61	60.30±14.72	0.09
Height (m)	1.57±0.10	1.59±0.11	1.56±0.10	0.26
SBP (mm/Hg)	136.73±24.29	138.13±22.78	135.33±26.02	0.11
DBP (mm/Hg)	83.27±13.55	84.80±12.63	81.73±14.47	0.23
BMI (kg/m^2^)	24.52±4.66	24.44±3.92	24.60±5.36	-0.03
BSA (m^2^)	2.19±0.55	2.23±0.50	2.15±0.60	0.14
Creatinine (mg/dL)	4.01±1.81	3.91±1.71	4.11±1.92	-0.11
CrCL (mL/min)	22.64±11.82	23.04±11.15	22.25±12.63	0.07
CrCL adjusted to Gender	20.32±10.16	20.78±9.90	19.86±10.56	0.09
eGFR (mL/min/1.73m^2^)	18.28±9.04	18.90±9.79	17.65±8.34	0.14
CKD grade (n)	Grade III: 7; grade IV: 28; grade V: 25	Grade III: 4; grade IV: 14; grade V: 12	Grade III: 3; grade IV: 14; grade V: 13	-0.10

The estimated marginal mean (EMM) in hemoglobin was 10.01±0.21 g/dL (95% CI: 9.59-10.4) at six months and increased from baseline of 7.99±0.73 g/dL in the desidustat arm and 9.89±0.21 g/dL (95% CI: 9.46-10.3) at six months from baseline in the erythropoietin arm (Table 2).

**Table 2:** Hematological parameters and biomarkers at six months (per-protocol analysis) Statistical test used: Between-group comparisons were performed using ANCOVA with baseline values as covariates. Values were expressed as mean±SD. Test statistic was reported as F-value (df=1). Effect size was reported as partial eta square. A p-value of <0.05 was considered statistically significant. ANCOVA was used by controlling baseline variability (as covariate) and treatment as fixed effect. Before testing ANCOVA, data was tested for normality of residual, homogeneity of variances, and independence of covariate and treatment, and violations were recorded. Violation of assumption: ^a^normality of residual; ^b^homogeneity of variances; ^c^independence of covariate and treatment ANCOVA: analysis of covariance; Hb: hemoglobin; RBC: red blood cell; MCV: mean corpuscular volume; MCH: mean corpuscular hemoglobin; MCHC: mean corpuscular hemoglobin concentration; RDW: red cell distribution width; TSAT: transferrin saturation; TIBC: total iron-binding capacity; GDF-15: growth differentiation factor-15; IL-6: interleukin-6; SD: standard deviation; CI: confidence interval

Treatment arm	Desidustat (n=26)			Erythropoietin (n=25)			Partial eta square	F-value	P-value
	Baseline score (mean±SD)	Post-intervention score (mean±SD)	Estimated marginal mean (95% CI)	Baseline score (mean±SD)	Post-intervention score (mean±SD)	Estimated marginal mean (95% CI)			
Hb (g/dL)	7.99±0.73	10.06±1.19	10.01±0.21 (9.59-10.4)	7.68±0.84	9.84±0.99	9.89±0.21 (9.46-10.3)	0.01	0.1726	0.6797
Hematocrit (%)	25.52±2.36	30.38±3.65	30.3±0.60 (29-31.5)	24.60±3.68	29.02±3.30	29.1±0.61 (27.9-30.4)	0.05	1.6282	0.2081
RBC count (million/µL)	2.78±0.33	3.38±0.38	3.34±0.06 (3.20-3.48)	2.64±0.48	3.32±0.40	3.36±0.07 (3.22-3.50)	0.00785	0.0239	0.8779^a^
MCV (fL)	91.25±10.40	89.00±4.58	89.4±1.11 (87.1-91.6)	93.17±8.41	87.92±6.61	87.5±1.13 (85.3-89.8)	0.00988	1.2759	0.2643^a^
MCH (pg)	28.59±2.83	29.56±1.81	30±0.47 (29.1-31)	29.90±4.13	29.50±3.48	29±0.48 (28.1-30)	0.000579	2.0941	0.1544
MCHC (g/dL)	30.98±2.28	33.20±2.04	33.4±0.44 (32.5-34.2)	31.74±2.50	33.01±2.61	32.8±0.44 (31.9-33.7)	0.00183	0.6398	0.4277^a^
RDW (%)	15.01±1.84	14.21±1.57	13.7±0.25 (13.2-14.3)	14.77±1.84	14.84±1.19	14±0.25 (13.5-14.5)	0.00652	0.5627	0.4569
Reticulocyte count (%)	1.96±0.73	1.75±0.07	1.49±0.11 (1.26-1.72)	2.23±1.17	2.50	1.72±0.15 (1.4-2.04)	0.08	1.3933	0.2474
Serum iron (mcg/dL)	71.99±11.40	79.86±22.05	79.4±3.52 (72.3-86.6)	70.54±11.17	75.86±7.29	76.4±4.01 (68.3-84.5)	0.02	0.3214	0.5743^a^
Ferritin (ug/dl)	199.53±216.34	179.32±121.30	195±24.1 (146-244)	310.03±367.13	179.20±172.30	160±26.4 (106-214)	0.000000395	0.9517	0.3371^a^
TSAT (%)	20.30±4.16	29.03±25.68	29.0±4.21 (20.5-37.6)	20.01±3.00	23.33±3.47	23.3±4.79 (13.6-33.0)	0.02	0.8052	0.3755^a^
TIBC (ug/dl)	357.96±31.33	311.89±62.05	310±11.3 (287-333)	353.55±35.34	329.59±43.47	332±12.9 (306-358)	0.03	1.6407	0.2084^a^
GDF-15 (pg/mL)	577.3±375.07	799±466.46	816±54 (707-925)	613.1±551.73	916±588.3	898±55.1 (788-1009)	0.05	1.1378	0.2914^ab^
IL-6 (pg/mL)	92.3±144.02	68±109.79	49.5±5.64 (38.2-60.8)	38.6±43.75	20.6±26.1	39.8±5.75 (28.3-51.4)	0.43	1.3961	0.2432^ab^

**Figure 3:**
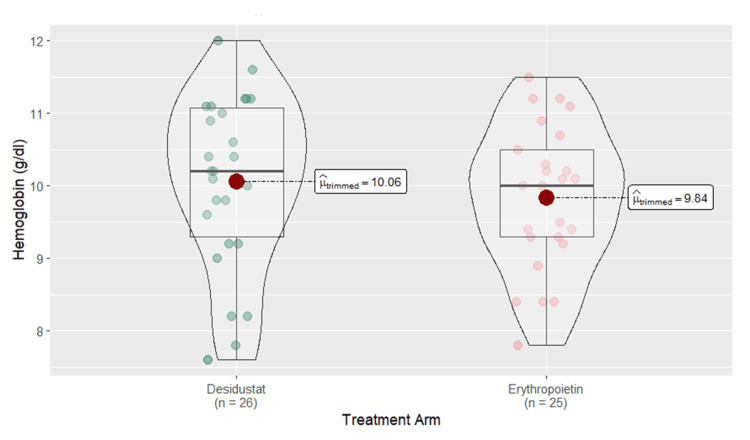
Box and violin plot comparing the hemoglobin of desidustat and erythropoietin arms at six months

The comparison displayed the results in a box and violin plot, showing the distribution of hemoglobin values for each arm. The mean hemoglobin was 10.06 g/dL for the desidustat arm (n=26; 86.7%) vs. 9.84 g/dL for the erythropoietin arm (n=25; 83.3%). No significant difference was found in the efficacy of both drugs in increasing hemoglobin levels between patients treated with desidustat and erythropoietin (p=0.47) (Figure 3).

The percentage of hemoglobin responders was higher in the desidustat arm (n=15; 57.69%) compared to the erythropoietin arm (n=12; 48%). There was no statistically significant difference (p=0.484) between the two arms at six months while comparing hemoglobin responders. Both treatments appear to have similar effects on correcting hemoglobin in this period (Table 3).

**Table 3:** Number of hemoglobin responder at six months (per-protocol analysis) The chi-square test was applied.

Study arm	Number of hemoglobin responder	Percentage	Pearson's chi-square	P-value
Desidustat (n=26)	15	57.69%	0.488	0.484
Erythropoietin (n=25)	12	48%		

**Figure 4:**
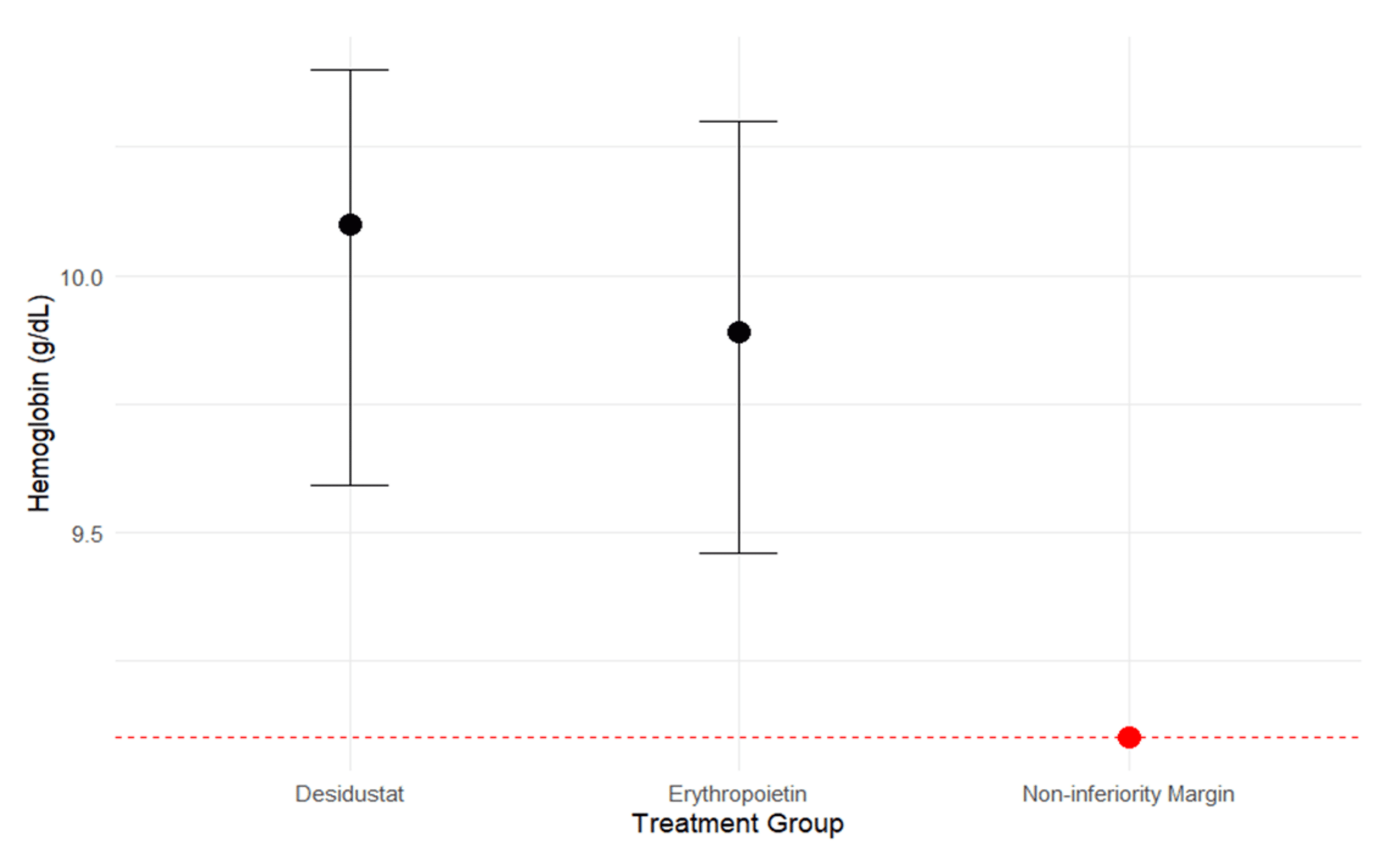
Non-inferiority plot comparing the mean hemoglobin between the two arms

Figure 4 represents the non-inferiority plot comparing the mean hemoglobin levels between the desidustat and erythropoietin arms after six months. The black dots represent the mean hemoglobin values, while the vertical bars indicate the range. The dashed red horizontal line denotes the predefined non-inferiority margin of 1 g/dL. The entire distribution of the desidustat arm was above this line, suggesting that desidustat was non-inferior to erythropoietin, as the lower bound of the 95% confidence interval for desidustat (9.59 g/dL) was greater than the erythropoietin EMM minus the non-inferiority margin (9.89-1.0=8.89 g/dL).

Both desidustat and erythropoietin showed comparable efficacy in improving red blood cell indices over six months, with no statistically significant difference between the two arms. According to our study, desidustat increased the mean serum iron (p=0.5743) and mean TSAT (p=0.3755) from baseline to six months and decreased the mean ferritin (p=0.3371) and mean total iron-binding capacity (TIBC) (p=0.2084) from baseline to six months, similar to that of erythropoietin. Overall, desidustat improved markers such as serum iron, TSAT, ferritin, and TIBC compared to those on erythropoietin. However, the results were not statistically significant, indicating that both treatments appeared to have similar effects on the iron profile, with no marked advantage of one over the other in this period (Table 2).

The mean IL-6 value for the desidustat arm was decreased from 92.3±144.02 pg/mL at baseline to EMM of 49.5±5.64 pg/mL (95% CI: 38.2-60.8), and for the erythropoietin arm, it was decreased from 38.6±43.75 pg/mL at baseline to EMM of 39.8±5.75 pg/mL (95% CI: 28.3-51.4) at six months. When comparing the difference between the arms, there was no statistically significant difference at six months (p=0.2432). The mean growth differentiation factor-15 (GDF-15) value for the desidustat arm was increased from 577.3±375.07 pg/mL at baseline to EMM of 816±54 pg/mL (95% CI: 707-925), and for the erythropoietin arm, it was increased from 613.1±551.73 pg/mL at baseline to EMM of 898±55.1 pg/mL (95% CI: 788-1009) at six months (p=0.291). When comparing the difference between the arms, there was no statistically significant difference at six months (Table 2).

**Figure 5:**
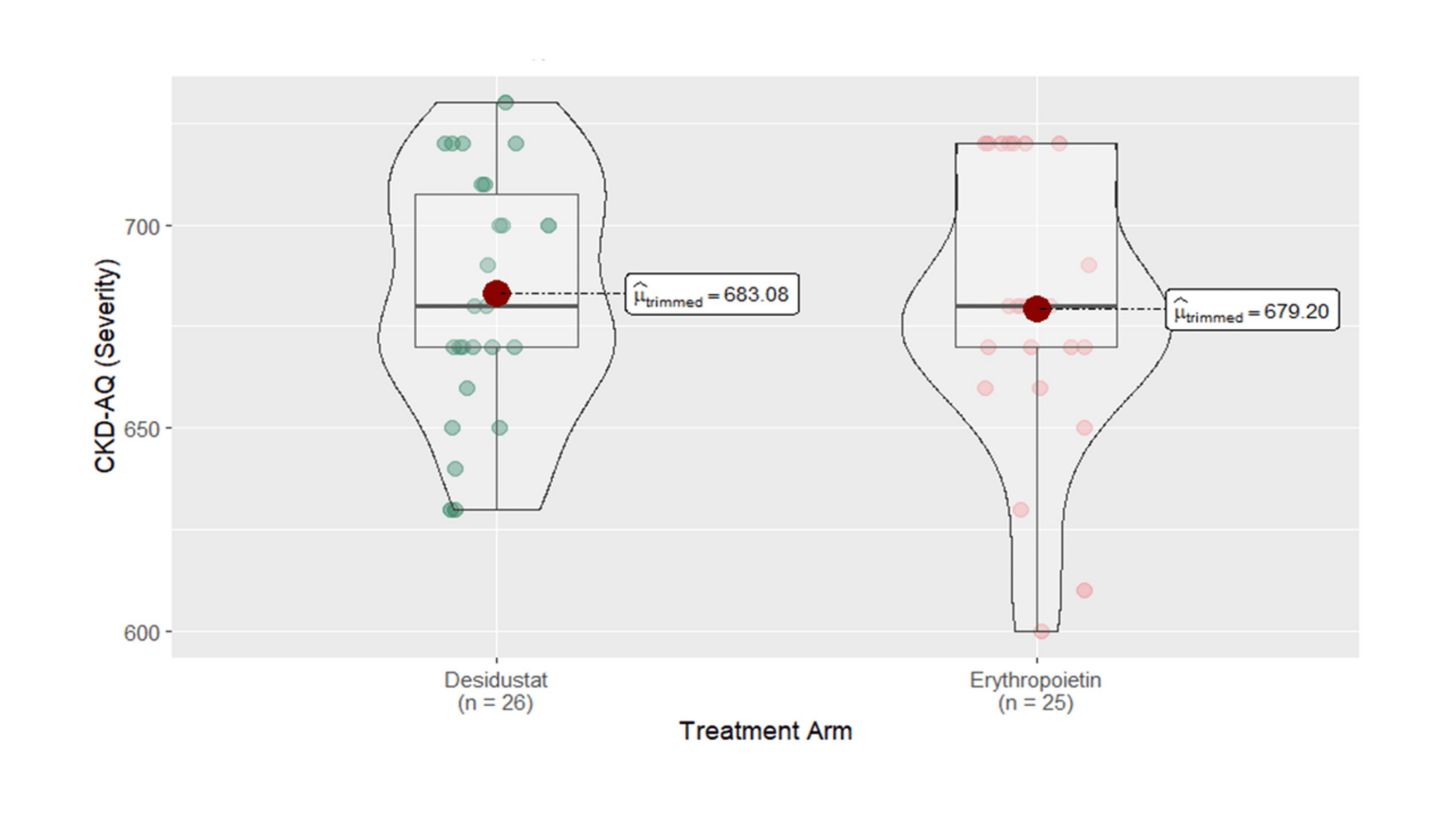
Box and violin plot comparing quality of life severity of desidustat and erythropoietin arms at six months CKD-AQ: Chronic Kidney Disease-Anemia Questionnaire [17] Permission for use was obtained from GlaxoSmithKline (GSK) via email. The link for the questionnaire (Mapi's ePROVIDE page link) is as follows: https://eprovide.mapi-trust.org/instruments/chronic-kidney-disease-anemia-questionnaire.

**Figure 6:**
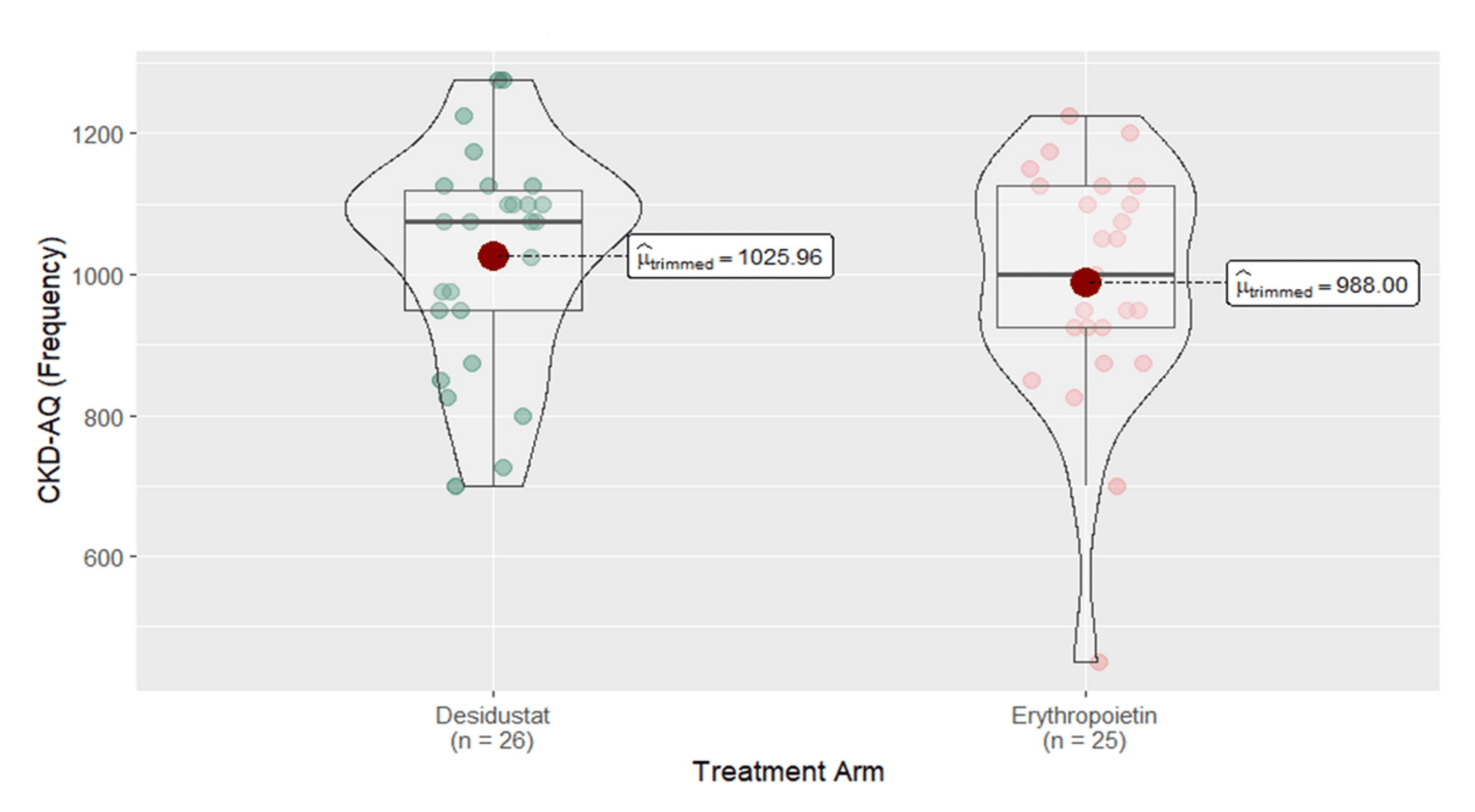
Box and violin plot comparing quality of life frequency of desidustat and erythropoietin arms at six months CKD-AQ: Chronic Kidney Disease-Anemia Questionnaire [17] Permission for use was obtained from GlaxoSmithKline (GSK) via email. The link for the questionnaire (Mapi's ePROVIDE page link) is as follows: https://eprovide.mapi-trust.org/instruments/chronic-kidney-disease-anemia-questionnaire.

The QoL score was assessed by CKD-AQ, which consisted of 23 items and was calculated by the severity score and frequency score. The mean severity score for the desidustat arm was increased from 303.33±44.82 at baseline to EMM of 596±43.2 (95% CI: 509-682) at six months, while for the erythropoietin arm, it was increased from 307.67±50.01 at baseline to EMM of 585±43.9 (95% CI: 497-673) at six months (p=0.8662). At each time point, desidustat had a greater improvement in severity scores compared to erythropoietin. The mean frequency score for the desidustat arm was increased from 215.83±112.28 at baseline to EMM of 882±69.6 (95% CI: 743-1021) at six months, while in the erythropoietin arm, it was increased from 162.5±112.33 at baseline to EMM of 867±70.8 (95% CI: 725-1009) at six months (p=0.8823). Desidustat demonstrated a larger increase in the frequency score compared to erythropoietin at each time point. Overall, the data indicate that the desidustat arm had higher severity and frequency scores compared to the erythropoietin arm, which reflected lesser symptom burden (Figures 5-6).

The results of three months of follow-up data of hematological parameters, iron profile, and QoL scores are shown in Table 4. Comparable results of desidustat and erythropoietin were observed in improving the abovementioned parameters over three months.

**Table 4:** Hematological parameters and quality of life scores at three months Statistical test used: Between-group comparisons were performed using ANCOVA with baseline values as covariates. Values were expressed as mean±SD. Test statistic was reported as F-value (df=1). Effect size was reported as partial eta square. A p-value of <0.05 was considered statistically significant. ANCOVA was used by controlling baseline variability (as covariate) and treatment as fixed effect. Before testing ANCOVA, data was tested for normality of residual, homogeneity of variances, and independence of covariate and treatment, and violations were recorded. Violation of assumption: ^a^normality of residual; ^b^homogeneity of variances; ^c^independence of covariate and treatment ANCOVA: analysis of covariance; Hb: hemoglobin; RBC: red blood cell; MCV: mean corpuscular volume; MCH: mean corpuscular hemoglobin; MCHC: mean corpuscular hemoglobin concentration; RDW: red cell distribution width; TSAT: transferrin saturation; TIBC: total iron-binding capacity; SD: standard deviation; CI: confidence interval

Treatment arm	Desidustat (n=30)			Erythropoietin (n=29)			Partial eta square	F-value	P-value
	Baseline score (mean±SD)	Post-intervention score (mean±SD)	Estimated marginal mean (95% CI)	Baseline score (mean±SD)	Post-intervention score (mean±SD)	Estimated marginal mean (95% CI)			
Hb (g/dL)	7.99±0.73	9.36±1.52	9.25±0.22 (8.8-9.7)	7.68±0.84	8.84±1.11	8.95±0.22 (8.49-9.41)	0.05	0.8659	0.3561^a^
Hematocrit (%)	25.52±2.36	28.61±4.33	28.3±0.65 (27-29.7)	24.60±3.68	27.18±3.83	27.5±0.67 (26.1-28.8)	0.04	0.8507	0.3603^a^
RBC count (million/µL)	2.78±0.33	3.18±0.56	3.1±0.08 (2.96-3.3)	2.64±0.48	3.08±0.54	3.13±0.08 (2.96-3.3)	0.01	0.0001	0.9909^a^
MCV (fL)	91.25±10.40	90.65±7.27	91.2±1.35 (88.4-93.9)	93.17±8.41	89.35±10.25	88.8±1.38 (86.1-91.6)	0.008	1.4562	0.2326
MCH (pg)	28.59±2.83	29.14±2.48	29.5±0.51 (28.5-30.5)	29.90±4.13	29.27±4.20	28.9±0.52 (27.8-29.9)	0.000579	0.675	0.4148
MCHC (g/dL)	30.98±2.28	32.69±2.01	32.8±0.34 (32.1-33.5)	31.74±2.50	32.50±2.15	32.4±0.35 (31.7-33.1)	0.0257	0.8827	0.3515
RDW (%)	15.01±1.84	14.58±1.25	14.5±0.20 (14.1-14.9)	14.77±1.84	14.32±1.51	14.5±0.19 (14.1-14.9)	0.00183	0.0004	0.9847
Reticulocyte count (%)	1.96±0.73	2.40	1.95±0.09 (1.77-2.14)	2.23±1.17	1.07±0.32	2.01±0.11 (1.79-2.24)	0.03	0.1788	0.6752^b^
Serum iron (mcg/dL)	71.99±11.40	73.13±8.43	72.6±1.42 (69.7-75.5)	70.54±11.17	70.00±8.58	70.6±1.47 (67.6-73.6)	0.08	0.9387	0.3415
Ferritin (ug/dl)	199.53±216.34	155.08±165.17	208±39.6 (126-290)	310.03±367.13	274.23±271.18	221±39.6 (139-303)	0.17	0.0521	0.8215^a^
TSAT (%)	20.30±4.16	21.64±4.84	21.8±0.87 (20-23.6)	20.01±3.00	21.09±2.97	20.9±0.93 (19-22.8)	0.0069	0.5444	0.467^a^
TIBC (ug/dl)	357.96±31.33	345.47±48.17	334±9.21 (315-353)	353.55±35.34	334.50±39.09	347±9.56 (327-366)	0.03	0.8189	0.3738
Severity	303.33±44.82	543±64.06	543±11.8 (520-567)	307.67±50.01	521±119.38	539±12 (515-563)	0.08	0.0670	0.7967^a^
Frequency	215.83±112.28	614.16±175.51	599±28.8 (541-657)	162.5±112.33	509.17±179.97	542±29.3 (484-601)	0.08	1.8418	0.1802^a^

Safety analysis

No significant difference in occurrences of adverse events was found between both treatment arms. Adverse events experienced with desidustat were nausea, gastritis, pedal edema, epistaxis, and hypertension, while with erythropoietin were pain and injection site reaction, headache, weakness, tachycardia, hypertension, and apprehension. All treatment-emergent adverse events (TEAE) were mild and resolved on their own. No intervention was needed.

Correlation of improved hemoglobin profile with biochemical parameters and biomarkers

Pearson's correlation coefficients among various variables at six months were compared (N=51, all participants who completed the study), specifically hemoglobin difference, systolic blood pressure (SBP), diastolic blood pressure (DBP), body mass index (BMI), creatinine clearance (CrCL), blood urea nitrogen (BUN), calcium, phosphorus, sodium, potassium, albumin/globulin (A/G) ratio, bilirubin, alanine aminotransferase (ALT), GDF-15, and IL-6. Pearson's correlation coefficient (r) indicates the strength and direction of the linear relationship between the two variables. Comparing the hemoglobin difference with the abovementioned parameters, no significant correlation was found (Table 5).

**Table 5:** Correlation of hemoglobin difference with overall biochemical parameters and biomarkers Pearson's correlation was used to correlate hemoglobin difference with biochemical parameters and biomarkers. SBP: systolic blood pressure; DBP: diastolic blood pressure; BMI: body mass index; CrCL: creatinine clearance; BUN: blood urea nitrogen; A/G ratio: albumin/globulin ratio; ALT: alanine aminotransferase; GDF-15: growth differentiation factor-15; IL-6: interleukin-6; SD: standard deviation

Correlation with hemoglobin difference (N=51)	Mean±SD	Pearson's correlation (r)	P-value
SBP (mm/Hg)	135.5±23.6	0.001	0.996
DBP (mm/Hg)	82.7±13.4	0.216	0.127
BMI (kg/m^2^)	24.1±4.6	-0.213	0.134
CrCL (mL/min)	21.7±11	-0.084	0.559
BUN (mg/dL)	48.9±23.5	-0.152	0.286
Calcium (mg/dL)	9±0.6	0.056	0.698
Phosphorus (mEq/dL)	4.8±0.9	-0.235	0.097
Sodium (mEq/L)	139.1±4	-0.031	0.830
Potassium (mEq/L)	4.2±0.5	0.368^**^	0.008
A/G ratio	1.2±0.1	0.055	0.701
Bilirubin (mg/dL)	0.5±0.2	-0.227	0.109
ALT (U/L)	32.7±10.3	0.080	0.577
GDF-15 (pg/mL)	856.3±527.7	0.141	0.322
IL-6 (pg/mL)	44.7±83.2	0.121	0.399

## Discussion

In our study, patients received either oral desidustat or injected erythropoietin subcutaneously, with a standard of care treatment. Desidustat and erythropoietin showed comparable efficacy in improving hemoglobin levels. The mean hemoglobin level was found to increase at both the three-month and six-month time points. According to our study observations, desidustat was found to be non-inferior to erythropoietin in increasing and maintaining hemoglobin levels within the target range in patients with anemia due to CKD who were not on dialysis. Similar results were also found in the DREAM-ND, DREAM-D, and Pahari studies [13,18,19].

In addition to hemoglobin levels, we compared the impact of desidustat and erythropoietin on other hematological parameters over six months. Our study found that desidustat increased the mean hematocrit, mean red blood cell (RBC) count, mean mean corpuscular hemoglobin (MCH), and mean mean corpuscular hemoglobin concentration (MCHC) while decreasing the mean mean corpuscular volume (MCV) and mean red cell distribution width (RDW), mirroring the effects observed with erythropoietin. The increasing trends in MCH and MCHC observed with desidustat were similar to vadadustat [20].

The percentage of hemoglobin responders, in our study, was higher in the desidustat arm (15 (57.69%)) compared to the erythropoietin arm (12 (48%)), and the difference was not statistically significant (p=0.484), indicating that both treatments had comparable effects on hemoglobin correction over six months. This finding is in accordance with the Parmar et al., DREAM-ND, and DREAM-D studies [13,18,21]. The effectiveness observed in our study, regarding hemoglobin changes over time and the hemoglobin response rate, was also comparable to other HIF-PHIs such as roxadustat, molidustat, and vadadustat [20,22,23].

According to our study, desidustat demonstrated a significant impact on iron metabolism markers, showing an increase in the mean serum iron (p=0.5743) and mean TSAT (p=0.3755) from baseline to six months while also reducing the mean ferritin (p=0.3371) and mean TIBC (p=0.2084), comparable to the effects observed with erythropoietin. Notably, desidustat improved key markers such as serum iron, TSAT, ferritin, and TIBC effectively, suggesting its potential advantage in managing anemia in patients. This trend in iron profile changes with desidustat aligns with observations from the DREAM-ND study [18] and studies on other HIF-PHIs like roxadustat and vadadustat [20,22].

In our study, QoL assessed by CKD-AQ was a critical outcome in anemia management, reflecting the overall well-being of patients. Both desidustat and erythropoietin were associated with improvements in QoL scores. The desidustat arm had higher severity and frequency scores, indicating a lesser symptom burden or impact and better QoL outcomes compared to the erythropoietin arm. These findings align with the results from the DREAM-ND and DREAM-D studies, from baseline to weeks 12 and 24 [13,18].

The severity of adverse drug reactions (ADRs) was mild; hence, there was no need for discontinuation of therapy. The safety profile of desidustat and erythropoietin was comparable in our study, with both drugs being well-tolerated by the patients. The safety findings from the previous studies further support these observations [13,18,19].

GDF-15 belongs to the transforming growth factor-β cytokine superfamily. Concentration is higher in inflammation, acute injury, or cancer as well as illnesses associated with inefficient erythropoiesis. It has been suggested that the decreased production of hepcidin is triggered by high concentrations of GDF-15 and may have a role in maintaining iron homeostasis [24]. GDF-15 has potential nephroprotective functions attributed to both the downregulation of inflammation and the upregulation of nephroprotective factors that have anti-inflammatory properties [25].

Both treatments increased GDF-15 levels from baseline to six months without any statistically significant difference (p=0.2914). Farag et al. reported significant correlations between serum GDF-15 levels and key hematological parameters such as hemoglobin, ferritin, iron, and CRP [26]. However, Yoshida et al. found no continuous rise in GDF-15 levels with darbepoetin alfa or roxadustat treatment, and GDF-15 did not seem to influence hepcidin levels during roxadustat therapy [27].

In our study, IL-6 levels exhibited a significant decline over the six-month period in both arms. Specifically, when comparing the difference between the arms, erythropoietin decreased the level of IL-6 significantly at six months, compared to desidustat (p=0.2432). This reduction in IL-6 levels is indicative of an improved inflammatory status among the patients, aligning with previous research findings. Clinical trials reported that roxadustat can reduce hepcidin levels and may be effective in treating inflammation-induced anemia in CKD. Moreover, roxadustat has demonstrated anti-inflammatory and anti-fibrotic effects, where it delayed peritoneal fibrosis in a mouse model through the TGF-β/Smad signaling pathway [28].

We compared the hemoglobin difference from baseline to six months with biochemical parameters, as well as biomarkers. No correlation was found between hemoglobin difference, biochemical parameters, and biomarkers. No comparative study of HIF-PHI was found correlating the above parameters.

The major limitations of the study were the small sample size and the relatively short observation period of six months. The study with a small sample size was undertaken as it was a pilot study to assess the efficacy and safety. In our study, an open-label study was conducted, as the route of two medicines was different and a double-dummy study would have been very resource-intensive. A double-dummy model would have been better at controlling bias. Additionally, allocation concealment was not done, and randomization was performed by the treating physician, which may introduce selection bias. Future studies with longer follow-up may help in capturing the long-term outcomes of efficacy and safety.

## Conclusions

The study demonstrated that desidustat, a novel oral HIF stabilizer, was non-inferior to injectable erythropoietin in treating anemia in non-dialysis CKD patients over six months. Both desidustat and erythropoietin were found to be well-tolerated. Desidustat, with its convenient oral administration, may help in improving compliance and subsequently increase QoL. Hence, it may serve as an alternative to erythropoietin in the management of anemia in patients with dialysis-naïve CKD.
